# Rise of the human-mouse chimeric brain models

**DOI:** 10.1186/s13619-022-00135-6

**Published:** 2022-09-03

**Authors:** Peng Jiang, Mahabub Maraj Alam

**Affiliations:** Department of Cell Biology and Neuroscience, Rutgers University, 604 Allison Road, Piscataway, NJ 08854 USA

**Keywords:** Human pluripotent stem cells, Chimera, Macroglia, Astroglia, Oligodendroglia, Microglia, Neurons, Neurological Disorders

## Abstract

Human-mouse chimeras offer advantages for studying the pathophysiology of human cells in vivo. Chimeric mouse brains have been created by engrafting human fetal tissue- or pluripotent stem cell-derived progenitor cells into the neonatal mouse brain. This provides new opportunities to understand human brain development and neurological disorders.

## Background

Understanding disease pathogenesis and recapitulating species-specific disease mechanisms have been challenging in studying human neurological diseases. A lack of functional human brain tissues has led scientists to use human pluripotent stem cells (hPSCs) in developing models. By using hPSC-based in vitro 2-dimensional (2D) cell culture and 3D brain organoid models, basic aspects of the diseases, such as cell differentiation, migration, and neuron-glia interactions, can be examined. However, how these altered cellular events lead to the disruption of neural circuits under disease conditions remains to be studied. Ultimately, specific disease mechanisms can only be modeled in live animals to identify the links between the altered cellular functions and behavioral performance. Human-mouse chimeric brain models allow scientists to study the pathophysiology of human neural cells in vivo and elucidate the mechanisms of neurological diseases. Here we discuss the generation and application of human-mouse chimeric brain models for studying neurological diseases.

## Human-mouse chimeric glial and neuronal brain models

**Fig. 1:**
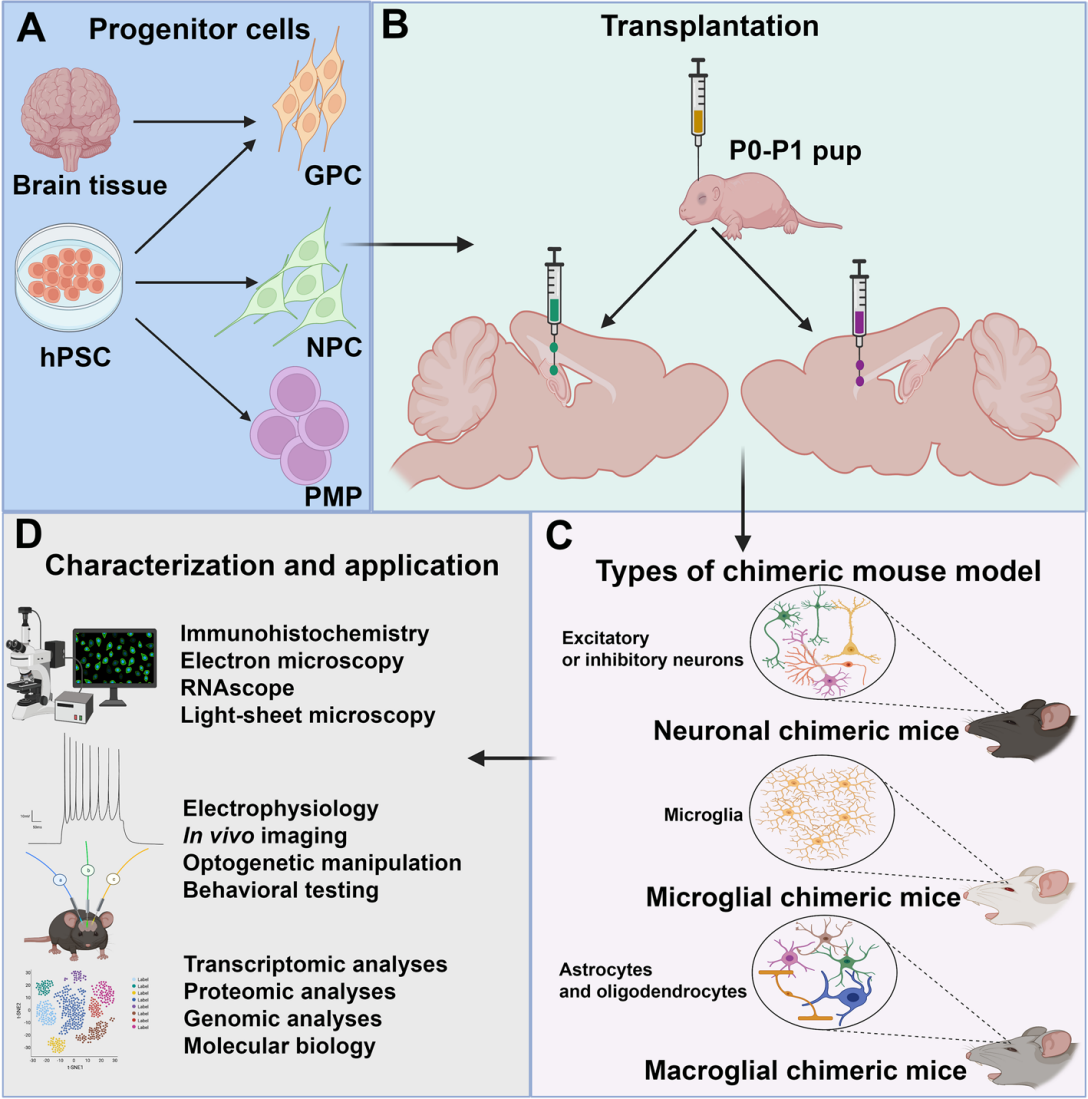
Schematic diagrams summarizing the generation, characterization, and application of the human-mouse chimeric brain models. **A** The progenitor cells, including glial progenitor cells (GPCs), primitive macrophage progenitors (PMPs), and neural progenitor cells (NPCs) are derived from human pluripotent stem cells (PSCs) or human fetal brain tissue. **B** Progenitor cells are engrafted to appropriate injection sites in neonatal immunodeficient mouse brain, such as the lateral ventricles, frontal cortex, hippocampus, as well as anlagen of the corpus callosum. **C** Three types of chimeric mouse models are established, including macroglial chimeric mice, microglial chimeric mice, and neuronal chimeric mice, in which the brain is widely populated by human astroglia and oligodendroglia, human microglia, and human excitatory or inhibitory neuron, respectively. **D** Characterization and application of chimeric mouse models using a variety of techniques

Human-mouse chimeric brains can be created by implanting human glial progenitor cells (GPCs), primitive macrophage progenitors (PMPs), or neural progenitor cells (NPCs) into neonatal mouse brains at selected anatomical sites (Fig. 1). The extraordinary ability of human cells to widely disperse and functionally integrate in the adult mouse brain indicates a level of conserved fundamental biological mechanisms involved in neural cell and circuit development, which form the basis of the human-mouse chimeric brain models.

One of the first effective brain chimerism was originally achieved by transplantation of human fetal brain tissue-derived glial progenitor cells (GPCs). Goldman and colleagues demonstrate that engrafted human fetal brain tissue- or hPSC-derived GPCs differentiate to macroglia (astroglia and oligodendroglia) in the mouse brain (Windrem et al. 2008). Engrafted human GPCs (hGPCs) can continue to divide for up to one year post-transplantation, thus, dispersal of human cells is extensive and involves various brain regions, generating human macroglial mouse chimeric brain. Over time, human glia cells gradually replace their host counterparts, due to the fact that hGPCs divide at rates substantially higher than those of endogenous murine progenitors (Han et al. 2013, Windrem et al. 2014). The hGPCs respond to signaling cues from the host brain; for example, in hypomyelinated immunodeficient *Shiverer* mice, hGPCs differentiate with a bias towards oligodendroglia (Windrem et al. 2017, Windrem et al. 2014), whereas in myelin wildtype immunodeficient mice, hGPCs differentiate more towards astroglia (Han et al. 2013, Osipovitch et al. 2019, Windrem et al. 2014). Once engrafted, cells are able to survive throughout the life span of the recipient mice and can be found up to 20 months post-implantation(Han et al. 2013). In chimeric *Shiverer* brains, hGPCs produce human oligodendrocytes, which ensheath murine axons and form compact myelin (Windrem et al. 2017). Neonatally transplanted hGPCs can also remyelinate axons in the adult mouse brain following cuprizone treatment (Windrem et al. 2020). Several myelinogenesis pathways in these cuprizone-treated hGPCs were not described in rodents before, further highlighting the relevance of chimeric models in recapitulating human disease conditions and developing stem cell regenerative medicine. Human astroglia derived from engrafted hGPCs integrate structurally and functionally within the host tissue while retaining their complex morphology and primate-specific interlaminar astrocytes, suggesting that astroglial human-specific features are cell-intrinsic and can be recapitulated in chimeric brains (Han et al. 2013). These human astroglia enhance learning in adult chimeric mice via release of TNF-α (Han et al. 2013).

Recent advances in stem cell differentiation technologies have led to the generation of microglial chimeric mouse model, in which hPSC-derived PMPs are implanted. Xenografted PMPs differentiate and mature into microglia that retain complex region-specific phenotypic and functional characteristics of human microglia (reviewed in (Jiang et al. 2020)). Microglia have been implicated in neurodevelopmental and neurodegenerative neurological disorders (Prinz et al. 2021). Transplanted human microglia with TREM2 deletion (TREM2KO) – an Alzheimer’s disease (AD) risk locus – in AD mouse models respond to beta-amyloid pathology similarly to murine microglia (McQuade et al. 2020).Transplanted human TREM2KO microglia in AD mouse models remain homeostatic rather than transitioning to disease-associated microglia (DAM) subtype, but interestingly, the lost ‘DAM’ population partially overlaps with the murine counterpart in gene expression profile. Down syndrome (DS) is the most common genetic origin of intellectual disability and the most common risk factor for AD (Lott and Head 2019). In DS human induced pluripotent stem cell (hiPSC)-based microglial chimeric brains, DS microglia exhibit enhanced synaptic pruning function, resulting in impaired synaptic neurotransmission and plasticity (Jin et al. 2022). Upon being exposed to pathological tau proteins, DS microglia display dystrophic and senescent phenotypes, recapitulating microglial pathology seen in human brain tissue derived from AD patients. Further single-cell RNA-sequencing analyses of chimeric mouse brains and mechanistic studies demonstrate that inhibiting type I interferon signaling can correct DS microglia dysfunction and prevents senescence(Jin et al. 2022). These studies show that human-mouse microglial chimeric models can be used to study pathophysiology of human microglia during brain development and in neurodegenerative diseases. Moreover, human-mouse chimeric models can be used to study novel functional aspects of genes and variants expressed by human cells in vivo.

Human PSC-derived NPCs have been used to generate human-mouse neuronal chimeras, in which NPCs differentiate and widely populate the host brain with human neurons (Chen et al. 2016, Espuny-Camacho et al. 2017, Linaro et al. 2019, Xu et al. 2019). Donor-derived human excitatory neurons undergo prolonged morphological, electrophysiological, and synaptic maturation within the host brain and exhibit spine sizes consistent with human neurons (Linaro et al. 2019). These findings suggest that human neuron-specific development and phenotypes in the mouse brain are partly governed by cell-intrinsic mechanisms. Donor-derived human neurons functionally integrate into the mouse brain by forming synaptic contacts between human and host neurons (Chen et al. 2016, Espuny-Camacho et al. 2017, Linaro et al. 2019). Spontaneous and evoked postsynaptic neurotransmission have been identified in engrafted human neurons (Linaro et al. 2019, Xu et al. 2019). Some human neurons in the mouse visual cortex exhibit visual-stimuli-driven calcium transients and show host-neuron-like specificity for stimulus direction or orientation (Linaro et al. 2019). When transplanted into the neonatal brain of a murine AD model, donor NPC-derived human neurons showed neuropathological hallmarks of AD in the adult mouse brain and importantly, exhibited neuronal cell death, which was absent in the host mouse neurons (Espuny-Camacho et al. 2017). Engrafted hiPSC-derived interneuron progenitor cells can differentiate and widely populate the mouse cerebral cortex with human interneurons. Mice transplanted with DS hiPSC-derived interneurons displayed impaired recognition memory compared to control mice (Xu et al. 2019). These findings support the notion that development, maturation, and aging of transplanted human cells can be influenced by the host brain environment. In turn, the human cells can structurally integrate and modulate the function of host brain circuitry and further impact animal behavior.

## Conclusions

Human-mouse chimeric brain models permit studies of: *i)* the development, integration, and function of human neural cells in vivo; *ii)* aging and neurodegeneration of engrafted human neural cells; *iii)* whether engrafted “diseased” human cells alter neural circuit formation and synaptic plasticity, and cause behavioral changes; and *iv)* whether modulating expression of genes could rescue “diseased” cells’ phenotypes. Future improvement in chimeric models could include generating chimeric brains containing multiple types of human neural cells and chimeric brains together with humanized immune systems (Jiang et al. 2020, Jin et al. 2022). The human-mouse chimeric brain models present possibilities for augmenting our understanding of human brain development and pathology.

## Data Availability

Not applicable.

## References

[CR1] Chen C, Kim WY, Jiang P (2016). Humanized neuronal chimeric mouse brain generated by neonatally engrafted human iPSC-derived primitive neural progenitor cells. JCI Insight.

[CR2] Espuny-Camacho I, Arranz AM, Fiers M, Snellinx A, Ando K, Munck S (2017). Hallmarks of Alzheimer's Disease in Stem-Cell-Derived Human Neurons Transplanted into Mouse Brain. Neuron..

[CR3] Han X, Chen M, Wang F, Windrem M, Wang S, Shanz S (2013). Forebrain engraftment by human glial progenitor cells enhances synaptic plasticity and learning in adult mice. Cell Stem Cell.

[CR4] Jiang P, Turkalj L, Xu R (2020). High-Fidelity Modeling of Human Microglia with Pluripotent Stem Cells. Cell Stem Cell.

[CR5] Jin M, Xu R, Wang L, Alam MM, Ma Z, Zhu S (2022). Type-I-interferon signaling drives microglial dysfunction and senescence in human iPSC models of Down syndrome and Alzheimer's disease. Cell Stem Cell..

[CR6] Linaro D, Vermaercke B, Iwata R, Ramaswamy A, Libe-Philippot B, Boubakar L (2019). Xenotransplanted Human Cortical Neurons Reveal Species-Specific Development and Functional Integration into Mouse Visual Circuits. Neuron..

[CR7] Lott IT, Head E (2019). Dementia in Down syndrome: unique insights for Alzheimer disease research. Nat Rev Neurol.

[CR8] McQuade A, Kang YJ, Hasselmann J, Jairaman A, Sotelo A, Coburn M (2020). Gene expression and functional deficits underlie TREM2-knockout microglia responses in human models of Alzheimer's disease. Nat Commun.

[CR9] Osipovitch M, Asenjo Martinez A, Mariani JN, Cornwell A, Dhaliwal S, Zou L (2019). Human ESC-Derived Chimeric Mouse Models of Huntington's Disease Reveal Cell-Intrinsic Defects in Glial Progenitor Cell Differentiation. Cell Stem Cell..

[CR10] Prinz M, Masuda T, Wheeler MA, Quintana FJ (2021). Microglia and Central Nervous System-Associated Macrophages-From Origin to Disease Modulation. Annu Rev Immunol.

[CR11] Windrem MS, Schanz SJ, Guo M, Tian GF, Washco V, Stanwood N (2008). Neonatal chimerization with human glial progenitor cells can both remyelinate and rescue the otherwise lethally hypomyelinated shiverer mouse. Cell Stem Cell.

[CR12] Windrem MS, Schanz SJ, Morrow C, Munir J, Chandler-Militello D, Wang S (2014). A competitive advantage by neonatally engrafted human glial progenitors yields mice whose brains are chimeric for human glia. J Neurosci.

[CR13] Windrem MS, Osipovitch M, Liu Z, Bates J, Chandler-Militello D, Zou L (2017). Human iPSC Glial Mouse Chimeras Reveal Glial Contributions to Schizophrenia. Cell Stem Cell..

[CR14] Windrem MS, Schanz SJ, Zou L, Chandler-Militello D, Kuypers NJ, Nedergaard M (2020). Human Glial Progenitor Cells Effectively Remyelinate the Demyelinated Adult Brain. Cell Rep.

[CR15] Xu R, Brawner AT, Li S, Liu JJ, Kim H, Xue H (2019). OLIG2 Drives Abnormal Neurodevelopmental Phenotypes in Human iPSC-Based Organoid and Chimeric Mouse Models of Down Syndrome. Cell Stem Cell..

